# Nurses’ intention to care of COVID-19 patients in hospitals dedicated to infectious disease in South Korea: application of the theory of planned behavior and verification of the moderating effect of ethical nursing competence

**DOI:** 10.1186/s12912-024-02072-y

**Published:** 2024-06-18

**Authors:** Mira Mo, Seongmi Moon, Eun Kyeung Song

**Affiliations:** 1 Ulsan University Hospital, 25 Daehakbyeongwon-ro, Dong-gu, Ulsan, 44033 South Korea; 2 Department of Nursing, College of Medicine, University of Ulsan, 93 Daehak-ro, Nam-gu, Ulsan, 44610 South Korea

**Keywords:** Nurses, COVID-19, Intention, Patient care, Ethics

## Abstract

**Background:**

The theory of planned behavior is a conceptual framework of recent studies to identify and explain nurses’ intentions to care for patients with emerging infectious diseases. However, correlations between behavioral intentions and variables that explain them have been inconsistent in previous studies. The influence of new variables might be considered in this case. This study aimed to determine moderating effects of ethical nursing competence on nurses’ intention to care for COVID-19 patients in hospitals dedicated to infectious diseases based on the theory of planned behavior.

**Methods:**

A cross-sectional survey was conducted. Data on intention to care for COVID-19 patients, perceived behavioral control, attitude toward the behavior, subjective norm, and ethical nursing competence were obtained from 190 nurses in three hospitals dedicated to infectious diseases in South Korea. The moderating effect of ethical nursing competence was analyzed using model I of PROCESS Macro. Ethical considerations: This study was approved by the Institutional Review Board of Ulsan University Hospital, South Korea. Written informed consent was obtained from each subject.

**Results:**

The ethical nursing competence was a significant moderator in the relation between perceived behavioral control and the intention to care (B = 0.36, t = 2.16, *p* = 0.032). Ethical nursing competence did not have a significant interaction with attitude toward behavior or subjective norm.

**Conclusions:**

This study showed that the higher the ethical nursing competence level, the greater the effect of perceived behavioral control on nurses’ intention to care for COVID-19 patients. Promoting ethical nursing competence is necessary for nurses who would take care of patients at the frontline of the infectious disease pandemic. Nursing managers should include ethical nursing competence in the assessment of nurses’ competence and design educational programs to enhance ethical nursing competence for efficient nursing staffing during a pandemic.

## Background

Due to the pandemic of infectious diseases caused by Coronavirus Disease 2019 (COVID-19), the Korean government has designated hospitals dedicated to infectious diseases for treating patients infected with COVID-19. As a result, many COVID-19 patients were sent to hospitals dedicated to infectious diseases. Proportion of COVID-19-related tasks and roles of nurses are increased. The work experience of nurses caring for COVID-19 patients in Korea shows that fear and concern about the spread of infection coexist with responsibility for patient care [1]. From the beginning of the 2020 COVID-19 pandemic to this point, nurses caring for patients with COVID-19 have had difficulty caring for patients due to limited information, unpredictable work, new challenges, insufficient support, family concerns, and emotional stress [2].

In a disaster caused by disease, the public expects healthcare providers to respond at the frontline. The most important ethical decision for healthcare providers at this time is whether to remain in hospitals to fight infectious diseases, which is a matter for each healthcare provider to decide for themselves [3]. After the COVID-19 pandemic, many research studies have been conducted on ethical issues that nurses face. An integrated analysis of these studies shows that nurses are required to work in situations where person protection equipment is scarce with their health endangered. They also experience moral distress due to the need to provide safe and high-quality care for patients [4]. Therefore, nurses’ intention to care for COVID-19 patients at hospitals dedicated to infectious diseases is important for ethical decision-making of nurses.

Nurses’ intention to care refers to their intention to perform nursing activities independently according to nurses’ intention to care for patients. In a situation where there are possibilities of emerging infectious diseases pandemic such as COVID-19, it is important to identify nurses’ intention to care [5]. Among theories that explain intentions to behavior, the theory of planned behavior (TPB) [6] is the conceptual framework of recent studies [5, 7–10] to identify and explain nurses’ intentions to care for patients with emerging infectious diseases. According to the TPB, perceived behavioral control (PBC), attitude toward the behavior (ATT), and subjective norm (SN) are three factors that can predict behavior intention [6]. In one study [5], all these three factors had a significant effect on the intention to care for patients with infectious diseases. However, there were cases in which ATT was not significant [8] or SN was not significant [8, 9]. In addition, one study [10] that investigated correlations among variables of TPB in nursing students reported that only PBC had a significant relationship with intention to care. As such, when correlations between behavior intention and variables that explain it are not consistent in previous studies, the influence of a new variable can be considered. In particular, the moderating action of the new variable can be verified [11]. The moderating action of the new variable could influence the direction or intensity of the relationship between PBC, ATT, and SN and intention to care. This means that the relationship varies according to the level of the new variable [11].

As mentioned earlier, when nurses’ intention to care for COVID-19 patients in a hospital dedicated to infectious diseases involves ethical decision-making, it can be thought that the influence of factors related to intention to care will vary depending on ethical decision-making abilities of nurses. According to a study [12] that investigated whether ethical characteristics of nurses could have a moderating effect in the model explaining nurses’ behaviors when the level of ethical leadership of nurses was high, the intention of eco-friendly behavior had a significant effect on actual eco-friendly behavior. In this study, ethical leadership showed a significant moderating effect when nurses’ intention to eco-friendly behavior was linked to actual eco-friendly behavior. Since nurses’ ethical leadership showed a moderating effect on the behavior- intention to behavior relationship, it is necessary to examine whether ethical characteristics could act as moderating variables in the relationship between PBC, ATT, and SN and intention to care based on TPB.

It is possible to add a new factor predicting intention to TPB. This predictive factor should be a factor that is conceptually independent of existing factors and a factor that can be widely applied to behavioral research conducted in social behavior science [13]. Ethical nursing competency, a concept that includes ethical decision-making of nurses, is a concept in which not only ethical decision-making but also ethical sensitivity, knowledge, reflection, behavior, and attitude are interdependently integrated. It develops in various ways depending on an individual’s situation regarding ethical problems and dilemmas [14]. Therefore, this study intends to apply TPB to explain nurses’ intentions to care for COVID-19 patients in hospitals dedicated to infectious diseases. The purpose of this study was to investigate the moderating effect of ethical nursing competency under the assumption that PBC, ATT, and SN have different effects on intention to care according to the level of ethical nursing competency. Results of this study suggest an expanded explanation of TPB and provide basic data for recognizing the importance of ethical nursing competency development of nurses by confirming the moderating effect of ethical nursing competency.

## Conceptual framework

**Fig. 1:**
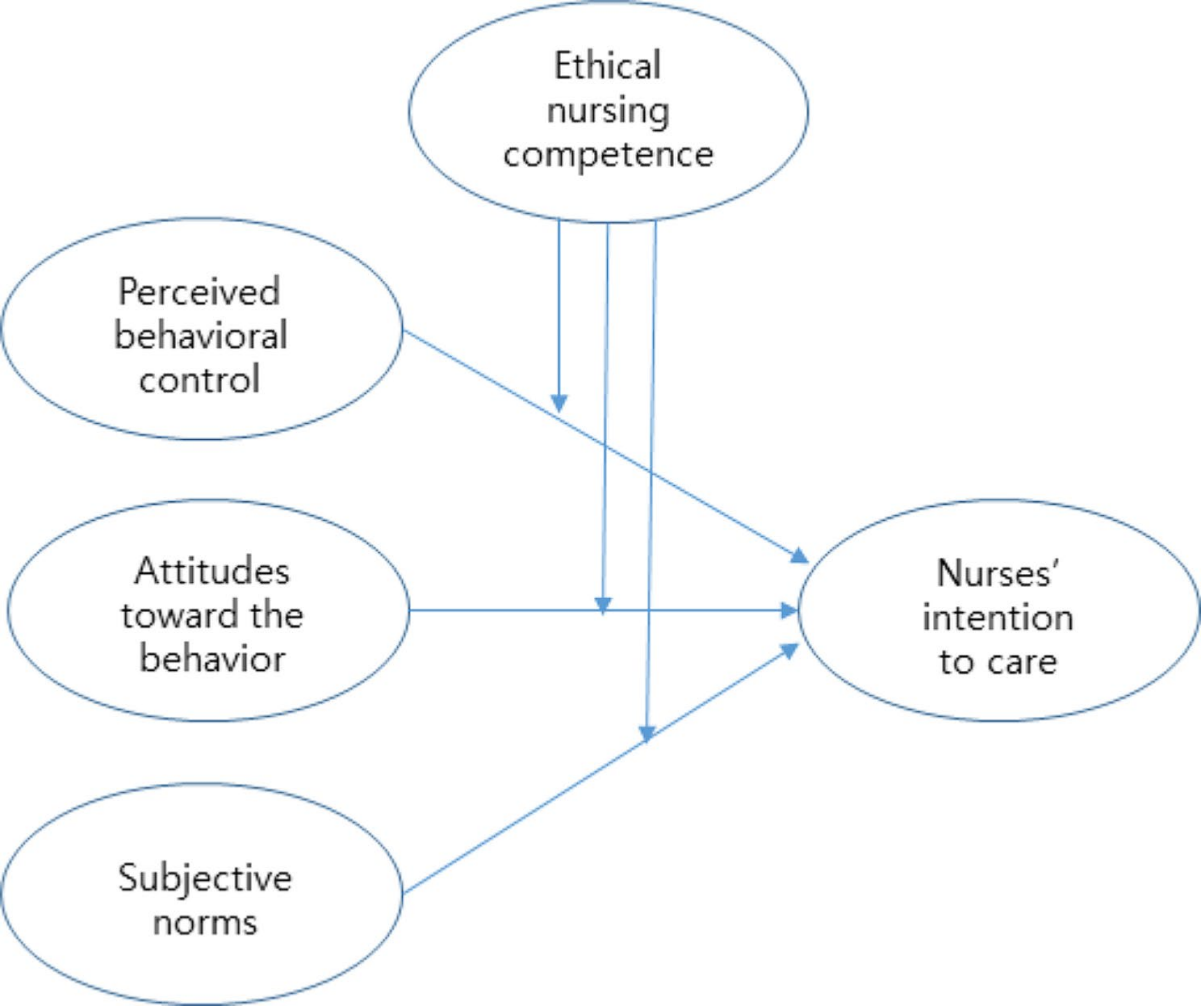
Conceptual framework of this study

In this study, we tried to confirm the moderating effect of ethical nursing competency based on TPB (Fig. 1) [6]. TPB is a theory proposed to predict complex human behavior and intention to behavior. PBC, ATT, and SN are key variables that can directly predict behavior intention. In this study, we will investigate whether ethical nursing competency can act as a moderating effect in the relationship between PBC-intention to care, ATT-intention to care, and SN-intention to care, respectively.

## Methods

### Design

This was a cross-sectional, correlational, descriptive study to identify the moderating effect of ethical nursing competency in the relationship between PBC, ATT, and SN and the care intention of nurses taking care of patients with COVID-19 at hospitals dedicated to infectious disease.

### Setting and sample

The participants in this study were nurses who worked at three general hospitals in the southeastern area of South Korea. These hospitals were specifically dedicated to infectious diseases. One hospital was situated in Ulsan Metropolitan City, while the other two were in Changwon City. Convenience sampling was used to select these hospitals. The study included nurses who had been directly caring for COVID-19 patients in isolated wards for more than 6 months. Nursing managers and nurses with administrative duties were not included in the study.

To calculate the sample size, regression analysis criteria were applied using the G*power program 3.1.9.2. At a significance level of 0.05 and a power of 90% with a median effect size and 15 predictors (11 general characteristics, 3 independent variables, and 1 moderating variable) as input, the required number of samples was calculated to be 171. Considering that previous studies have reported dropout rates of either 7% [5] or 8% [7], a sample of 190 nurses was conveniently recruited for this study, taking into account a projected dropout rate of 10%. All 190 questionnaires were analyzed.

### Measurements

#### TPB related variables

Instruments were developed to measure nurses’ intention to care, PBC, ATT, and SN in the care of patients with Severe Acute Respiratory Syndrome (SARS) based on the TPB [15]. These instruments were modified specifically for nurses caring for emerging infectious diseases. To ensure their validity, items were selected with high content validity index by a panel of five experts. This panel consisted of two nursing professors, one infection control nurse specialist, and two nurses with experience in caring for emerging infectious diseases [9].

We used the modified version. Instruments had a 7-point Likert-type rating from − 3 (strongly disagree) to 3 (strongly agree). We used the average value divided by the number of items by summing up scores of the items for each variable for analysis, with a higher score indicating a higher level of the variable.

Nurses’ intention to care was measured as a nurse’s willingness to care for COVID-19 patients using three items asking about their willingness to voluntarily perform nursing care. The Cronbach’s alpha in this study was 0.89.

The PBC consisted of two items asking whether a nurse felt confident in caring for COVID-19 patients and perceives ease in performing nursing care. The Cronbach’s alpha in this study was 0.87. The ATT consisted of three items asking whether caring for COVID-19 patients was wise, must-do, or worthwhile. The Cronbach’s alpha in this study was 0.83. The SN consisted of two items asking how much a nurse felt social pressure to care for patients with COVID-19. The Cronbach’s alpha in this study was 0.79.

#### Ethical nursing competency

Ethical nursing competency is a concept that interdependently integrates not only ethical decision-making, but also ethical sensitivity, knowledge, reflection, behavior, and attitude based on ethical knowledge and sensitivity in ethical conflict situations that occur during nursing work [14]. We used the ethical nursing competency measurement tool developed by Kang and Oh [16]. It consisted of a total of 20 items in five factors, including four items of ethical sensitivity, two items of ethical knowledge, two items of ethical reflection, six items of ethical decision-making and action, and six items of ethical behavior. The Cronbach’s alpha in this study was 0.88.

### Data collection

Data collection was conducted from August 10, 2021 to September 01, 2021. We reached out to the nursing departments of our target hospitals to provide them with an explanation of the purpose and methodology of our study. Once we received permission, the nursing department then notified the nurses about the survey through their respective nursing managers. To ensure maximum participation, we personally visited each department and handed out structured questionnaires to the nurses who willingly agreed to take part in the study. Once the questionnaires were completed, they were sealed and collected at the nursing department. We subsequently visited the nursing department to retrieve the questionnaires. In total, we collected 65 questionnaires from a hospital in Ulsan, and 85 and 40 questionnaires from two hospitals in Changwon, respectively.

### Data analysis

Data were analyzed using the SPSS 24.0 program. PROCESS Macro version 4.0 was used to analyze the moderating effect of ethical nursing competency. For general characteristics, frequency and percentage were calculated. Intention to care, PBC, ATT, SN, and ethical nursing competency were analyzed. Results are presented as mean and standard deviation. Differences in intention to care by participants’ general characteristics were analyzed using an independent t-test and one-way analysis of variance. Correlations between intention to care, PBC, ATT, SN, and ethical nursing competency were analyzed using Pearson’s correlation coefficient. To verify the moderating effect of ethical nursing competency, PROCESS Macro model 1 was used [17].

## Results

### General characteristics of participants

Table 1 shows general characteristics of participants. Of all participants, 86.3% were women, 85.3% were under 40 years old, 75.8% were unmarried, and 81.1% were staff nurses. Among these participants, 91.6% worked in wards and 8.4% worked in intensive care units. As for the number of patients in charge, 42.1% had 20 or less, and 39.5% had 40–60 patients. Among participants, 96.3% had experience in education related to infectious diseases, 71.6 had experience in education related to ethics, and 81.6% had no experience of caring for patients with emerging infectious diseases. Regarding perceived work intensity, ‘upper-middle’ was the most at 56.8%, followed by ‘upper’ at 25.3% (48 persons), ‘middle’ at 16.8%, and ‘middle-lower’ at 1.1%.

**Table 1:** General Characteristics of Subjects (*N* = 190)

Variables	Categories	n (%)	Intention to care	
			Mean ± SD	t/F (*p*)
Gender	Male	7 (3.7)	0.90 ± 1.51	-0.31 (0.757)
	Female	183 (96.3)	1.05 ± 1.20	
Age (yrs)	< 40	162 (85.3)	0.96 ± 1.13	-2.37 (0.019)
	≥ 40	28 (14.7)	1.54 ± 1.50	
Marital status	Single	144 (75.8)	1.02 ± 1.12	0.45 (0.657)
	Married	46 (24.2)	1.12 ± 1.46	
Education level	College degree	24 (12.6)	1.11 ± 1.34	0.52 (0.598)
	Bachelor’s degree	154 (8.1)	1.01 ± 1.16	
	Master’s degree or higher	12 (6.3)	1.36 ± 1.51	
Working department	Ward	174 (91.6)	1.06 ± 1.22	0.73 (0.467)
	ICU	16 (8.4)	0.83 ± 1.09	
Number of patients per duty per person	≤ 20	80 (42.1)	1.25 ± 1.09	2.16 (0.118)
	21 ~ 40	35 (18.4)	0.85 ± 1.31	
	41 ~ 60	75 (39.5)	0.91 ± 1.26	
Perceived work intensity	Upper	48 (25.3)	0.83 ± 1.36	0.68 (0.568)
	Upper-middle	108 (56.8)	1.10 ± 1.16	
	Middle	32 (16.8)	1.14 ± 1.14	
	Middle-lower	2 (1.1)	1.33 ± 0.94	
Position	Staff nurse	154 (81.1)	0.97 ± 1.17	-1.82 (0.071)
	Charge, head nurse	36 (18.9)	1.37 ± 1.33	
Completed infectious disease education	Yes	183 (96.3)	1.04 ± 1.22	-0.01 (0.993)
	No	7 (3.7)	1.05 ± 0.59	
Completed ethics education	Yes	136 (71.6)	1.09 ± 1.22	0.76 (0.448)
	No	54 (28.4)	0.94 ± 1.17	
Experience in nursing patients with emerging infectious diseases before COVID-19	Yes	35 (18.4)	1.25 ± 1.42	0.97 (0.336)
	No	155 (81.6)	1.00 ± 1.15	

ICU = Intensive Care Unit; SD = Standard deviation

### Intention to care, PBC, ATT, SN, and ethical nursing competency

Means and standard deviations of intention to care, PBC, ATT, SN, and ethical nursing competency are shown in Table 2.

**Table 2:** Description of Variables (*N* = 190)

Variables	Mean ± SD	Min	Max	*r* (*p*)			
				INT	PBC	ATT	SN
Intention to care	1.04 ± 1.21	-3.00	3.00	1			
Perceived behavioral control	0.86 ± 1.13	-3.00	3.00	0.50 (< 0.001)	1		
Attitude toward the behavior	1.44 ± 1.07	-3.00	3.00	0.51 (< 0.001)	0.41 (< 0.001)	1	
Subjective norm	0.99 ± 1.01	-2.00	3.00	0.35 (< 0.001)	0.49 (< 0.001)	0.37 (< 0.001)	1
Ethical nursing competence	3.07 ± 0.30	2.10	4.00	0.26 (< 0.001)	0.34 (< 0.001)	0.32 (< 0.001)	0.37 (< 0.001)
Ethical sensitivity	3.07 ± 0.40	2.00	4.00				
Ethical knowledge	2.96 ± 0.39	1.00	4.00				
Ethical reflection	2.72 ± 0.57	1.00	4.00				
Ethical decision making and action	3.09 ± 0.31	2.17	4.00				
Ethical behavior	3.20 ± 0.39	2.00	4.00				

ATT = Attitude toward the behavior; INT = Intention to care; PBC = Perceived behavioral control; SD = Standard deviation; SN = Subjective norm

### Differences in intention to care by participants’ general characteristics

Participants’ intention to care was significantly different by age. Intention to care for those in their 40s or older had a score of 1.54 ± 1.50, which was significantly higher than that (0.96 ± 1.13) in those under 40 years (t = -2,37, *p* = 0.019). In the case of charge nurses or head nurses, intention to care had a score of 1.37 ± 1.33, higher than that of staff nurses at 0.97 ± 1.17, although the difference was not statistically significant (t = 1.82, *p* = 0.071). For those working in general wards rather than intensive care units (t = 0.73, *p* = 0.467), for those whose number of patients in charge was relatively small (t = 2.16, *p* = 0.118), and for those who perceived work intensity as ‘middle-lower’ (t = 0.68, *p* = 0.568), their intention to care was higher, although there were no significant differences.

Intention to care was almost the same regardless of whether or not they had received education on infectious diseases. Intention to care was higher in those who had received education on ethics (t = 0.76, *p* = 0.448) and revious experience of caring for patients with emerging infectious diseases (t = 0.97, *p* = 0.336), although there were no statistically significant differences (Table 1).

### Relationships among Intention to care, PBC, ATT, SN, and ethical nursing competency

Intention to care was significantly related to PBC (*r* = 0.50, *p* < 0.001), ATT (*r* = 0.51, *p* < 0.001), SN (*r* = 0.35, *p* < 0.001), and ethical nursing competency (*r* = 0.26, *p* < 0.001) (Table 2).

### Moderating effect of ethical nursing competency

To construct a model of moderating effect of ethical nursing competency, the variable ‘age’ among general characteristics which had significant association with intention to care was included in the model (Table 3). In model 1 (F = 18.690, *p* < 0.001), in which ethical nursing competency played a moderating role in the relationship between PBC and intention to care, PBC affected intention to care only by its interaction with ethical nursing competency with a significant effect (t = 2.16, *p* = 0.032). ATT independently had a significant effect on intention to care (t = 4.81, *p* < 0.001). In model 2 (F = 17.61, *p* < 0.001), in which ethical nursing competency had a moderating effect on the relationship between ATT and intention to care, the interaction between ATT and nursing ethical competency was insignificant (t = 0.76, *p* = 0.451). Only PBC independently had a significant effect on intention to care (t = 4.51, *p* < 0.001). In model 3 (F = 17.48, *p* < 0.001), in which ethical nursing competency had a moderating effect on the relationship between SN and intention to care, the interaction between SN and ethical nursing competency was insignificant (t = 0.20, *p* = 0.843). PBC (t = 4.56, *p* < 0.001) and ATT (t = 5.12, *p* < 0.001) each independently had a significant effect on intention to care.

**Table 3:** Moderating Effect of Ethical Nursing Competence (*N* = 190)

Model	Variables	B	S.E	t	*p*	LLCI	ULCI
Model 1	PBC	-0.70	0.50	-1.42	0.158	-1.68	0.28
	ENC	-0.22	0.30	-0.75	0.457	-0.80	0.36
	PBC X ENC	0.36	0.17	2.16	0.032	0.03	0.69
	ATT	0.36	0.08	4.81	< 0.001	0.21	0.51
	SN	0.05	0.09	0.59	0.554	-0.12	0.23
	Age (≥ 40)	-0.18	0.21	-0.86	0.394	-0.61	0.24
	R²=0.38, F = 18.69, *p* < 0.001						
Model 2	ATT	-0.11	0.67	-0.16	0.565	-2.00	3.65
	ENC	-0.24	0.48	-0.50	0.619	-1.19	0.71
	ATT X ENC	0.17	0.22	0.76	0.451	-0.27	0.60
	PBC	0.35	0.08	4.51	< 0.001	0.20	0.51
	SN	0.07	0.09	0.83	0.406	-0.10	0.24
	Age (≥ 40)	-0.13	0.22	-0.60	0.553	-0.55	0.30
	R²=0.37, F = 17.61, *p* < 0.001						
Model 3	SN	-0.05	0.69	-0.08	0.938	-1.41	1.31
	ENC	0.01	0.39	0.02	0.983	-0.75	0.77
	SN X ENC	0.04	0.22	0.20	0.843	0.39	0.48
	PBC	0.36	0.08	4.56	< 0.001	0.20	0.51
	ATT	0.39	0.08	5.12	< 0.001	0.24	0.54
	Age (≥ 40)	-0.12	0.22	-0.56	0.574	-0.55	0.30
	R² = 0.36, F = 17.48, *p* < 0.001						

ATT = Attitude toward the behavior; B = unstandardized estimates; ENC = Ethical nursing competence; INT = Intention to care; LLCI = Low Limit Confidence Interval; PBC = Perceived behavioral control; SD = Standard deviation; S.E = Standardized Error; SN = Subjective norm; ULCI = Upper Limit Confidence Interval

**Fig. 2:**
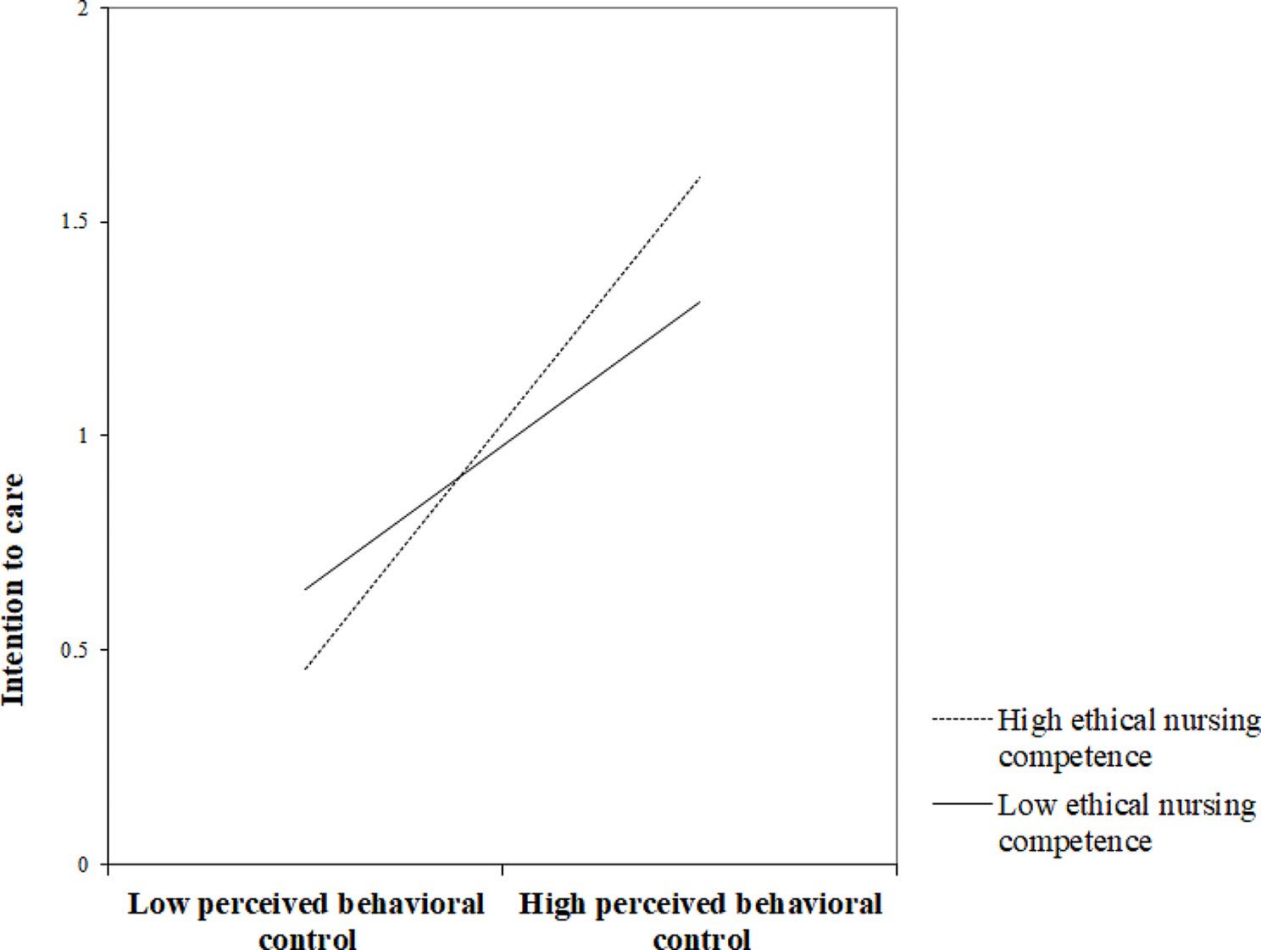
Effect of perceived behavioral control on intention to care at values of moderator ethical nursing competence

**Fig. 3:**
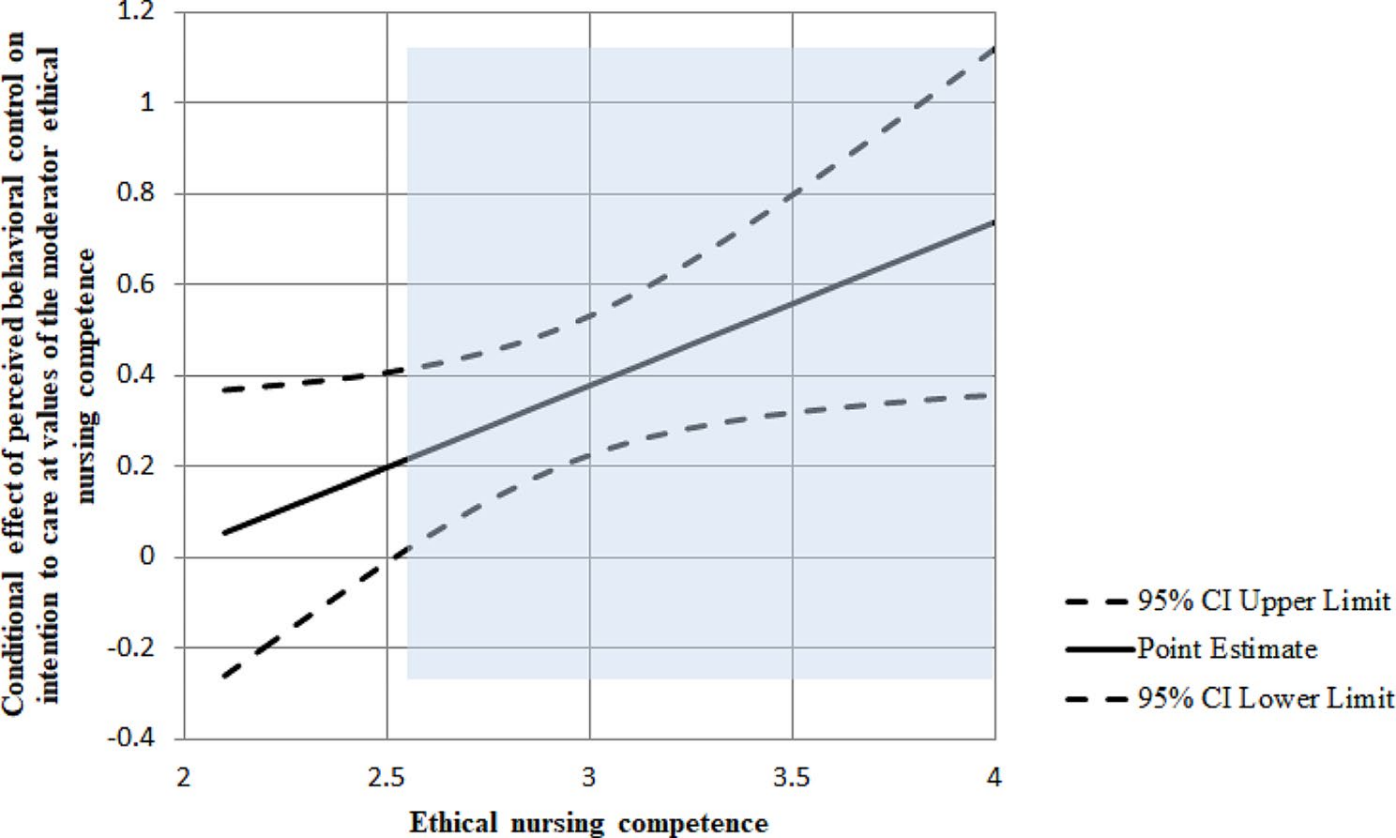
Conditional effect of perceived behavioral control on intention to care depending on moderator ethical nursing competence

In model 1, in which the moderating effect of ethical nursing competency was significant, the relationship between PBC and intention to care in the group with a low level of ethical nursing competency (-1 standard deviation) and the group with a high level (+ 1 standard deviation) according to the pick-a-point approach is shown in Fig. 2. In the case of the group with a low level of ethical nursing competency, the effect size of PBC affecting intention to care was 0.30. In the group with a high level of ethical nursing competency, the effect size was 0.51 (Table 4). In other words, the higher the ethical nursing competency, the greater the influence of PBC on intention to care. According to Johnson-Neyman analysis, the moderating effect of ethical nursing competency was significant when the ethical nursing competency exceeded 2.52 points (Fig. 3).

**Table 4:** Conditional Effects of Perceived Behavioral Control at Values of Ethical Nursing Competence

Moderating variable	Level	Effect	S.E	t	*p*	LLCI	ULCI
Ethical nursing competence	2.77 (-1 SD)	0.30	0.08	3.62	< 0.001	0.14	0.46
	3.07 (Mean)	0.40	0.08	5.03	< 0.001	0.25	0.56
	3.37 (+ 1 SD)	0.51	0.11	4.86	< 0.001	0.30	0.72

SD = Standard Deviation; S.E. = Standardized Error; LLCI = Low Limit Confidence Interval; ULCI = Upper Limit Confidence Interval

## Discussion

In this study, we tried to explain the intention to care of nurses who cared for COVID-19 patients in hospitals dedicated to infectious diseases. The intion to care had a score of 1.04 points. In a previous study [18] in which data were collected one year earlier than this study, the intention to care of nurses caring for patients with COVID-19 also had a score of 1.04 points. In studies conducted in Korea, nurses who had experience caring for COVID-19 patients during the COVID pandemic had a higher intention to care than nurses who did not [18, 19]. In addition, nurses’ intention to care during the COVID pandemic was higher than that of patients with new infectious diseases investigated before the pandemic [5, 9].

Ehtical nursing competency is as a comprehensive concept that reflects various ethical attributes, such as ethical knowledge, attitudes, sensitivity, reflection, decision-making, and behavior. It can be considered as a new concept that expands TPB to explain intention behind actions [13]. Among ethical issues that nurses experience and feel confusion and conflict in Korea, priorities are ‘patient care situations that can threaten nurses’ health’ and ‘staffing that restricts nursing care’. Accordingly, there is also a high demand for ethics education on situations that can threaten nurses’ health [20]. In the COVID-19 pandemic, nurses are experiencing ethical conflicts on a daily basis. Lack of understanding of COVID-19 transmission mode and characteristics, pathophysiology, and susceptibility profile along with insufficient personal protective equipment placed nurses at significant and uncertain risks. Nonetheless, nurses strive to balance their duty of benevolence with duty of caring for patients [21]. They believe that all patients have the right to receive optimal treatment regardless of age or health status [22]. Most nurses showed intention to participate in the care of patients with COVID-19 [23]. Although the level of stress is high while nursing patients with COVID-19, there is also a report that stress does not affect nursing intention because of professional ethics of nurses who are aware of their responsibility and obligation to care for patients [24]. In this study, these ethical characteristics of nurses were measured as ethical nursing competency. It showed a significant moderating effect in the relationship between PBC and intention to care in TPB. At this time, PBC itself did not influence the intention to care significantly. Results of this study confirm that despite perceived confidence and ease in caring for COVID-19 patients, the level of ethical nursing competence determines the extent to which PBC influences intention to care, ultimately demonstrating the importance of ethical nursing competence in determining intention to care.

Several studies have shown moderating effects of ethical factors in models explaining behaviors. One study has shown a moderating effect of ethical leadership on the relationship between nurses’ intention to engage in green behavior and actual green behavior [12]. In the field of management, researches have shown that the impact of public service motivation on organizational commitment is greater when there is a higher ethical leadership [25]. In addition, groups with high ethical leadership experienced an increased task commitment as cooperation increases, while groups with low ethical leadership experienced decreased task commitment even when cooperation increased [26]. Moderators are variables that can modify the strength or direction of a causal relationship. They are characterized as relatively stable personal characteristics or relatively unchanging environmental variables [27]. Further research is needed to determine whether ethical nursing competencies, which are ethical characteristics of nurses, such as ethical leadership, could serve as moderators in explaining nurses’ behavioral intentions and behaviors. Furthermore, the development and practical application of education and training programs to promote ethical nursing competence are needed.

Among variables presented in the TPB in this study, SN was the only variable that did not have a significant effect on the intention to care for COVID-19 patients. SN was 0.99, which was slightly higher than tjpse in previous studies [8, 19]. In Korean studies explaining the intention to care for patients with infectious diseases, including this study, results of no significant effect of SN [8, 9, 19] are more common than the results of significant effects [5, 7]. SN is known to be the variable least related to intention in the TPB [28, 29]. SN refers to the degree to which a person feels social pressure. There has been a consistent need to redefine this concept because social pressure is not so direct or explicit [28]. However, for behaviors that most people perceive as clearly necessary, such as vaccination and social distancing during the COVID-19 pandemic, SN has been shown to significantly influence behavior intentions [30, 31].

In the TPB, PBC has been studied as a variable that independently affects intention to behavior. According to Ajzen [13], PBC theoretically plays a moderating role in relationships between ATT- behavior intentions and SN-behavior intentions. In one study to identify this aspect [29], PBC was used as a moderating variable to explain intentions for three behaviors: voting, reducing food waste, and reducing energy consumption, with interaction effects of PBC and SN being found to be consistent. Given these results, the importance of SN is that it interacts with PBC to influence behavior intention. In this study, PBC is treated as an independent variable as it has been widely studied in previous studies. In this study, PBC was 0.86, which was high compared to previous studies [8, 19]. Models 2 and 3, which examined the interaction of ethical nursing competence with ATT and SN, showed that higher levels of PBC were associated with higher intention to care. Previous studies have consistently shown that PBC could independently influence intention to care [5, 7–9, 19]. On the other hand, in this study, PBC had a significant interaction effect with ethical nursing competence. As suggested by Ajzen [13], to verify the moderating role of PBC, it is necessary to continue to explore interactions between PBC and other variables in future studies.

According to the TPB, ATT can influence behavior intention. In this study, ATT was 1.50, which was similar to or higher than those in previous studies [8, 19]. Also, model 1 and model 3 used to examine the interaction of ethical nursing competence with PBC and SN found that more positive ATT was associated with a higher intention to care. In studies exploring the experience of caring for patients with COVID-19, nurses report feeling supported, recognized, respected, and proud by those around them and the public, which has led to a greater sense of mission and pride [1, 32–35]. A systematic review of qualitative studies on nurses’ experiences working in acute care hospitals during the respiratory pandemic [36] has found that nurses are willing to work together because they experience a sense of professional camaraderie, caring for and protecting their colleagues by sharing tasks. It can be inferred that the support, respect, recognition, and professional camaraderie that society has shown for nurses since the pandemic have led to an attitude that caring for COVID-19 patients is a worthwhile endeavor, which might have increased their intention to care. However, in this study, ethical nursing competencies did not have a significant moderating effect on the relationship between ATT and intentions to care, i.e., ATT influenced intentions to care regardless of the level of ethical nursing competencies. Thus, to increase intentions to care for COVID-19 patients, it is necessary for society to recognize and appreciate nurses’ contributions [23] as well as for nurses themselves to value their behaviors through supportive words from nursing managers [37] and financial rewards [9, 37].

Because this study included nurses working in infectious disease hospitals, it is important to be cautious when applying findings of this study to nurses who are not caring for patients with COVID-19 or have never cared for patients with COVID-19. In addition, this study used a convenience sample from three hospitals. Thus, caution should be exercised in generalizing results.

## Conclusions

This study extended the TPB to include ethical nursing competence as a moderating variable to explain nurses’ intention to care for COVID-19 patients in an infectious disease hospital. Nurses’ intention to care for COVID-19 patients was high. Ethical nursing competence had a significant moderating effect on the relationship between PBC and intention to care: the higher the level of ethical nursing competence, the greater the effect of PBC on intention to care. Based on findings of this study, it is necessary to explore various ways to enhance ethical nursing competence for nurses on the frontline of the emerging infectious disease pandemic.

## Data Availability

The data and materials of this study are available from the corresponding author upon reasonable request.

## Abbreviations

ATT: Attitude toward the behavior
COVID-19: Coronavirus Disease 2019
PBC: Perceived behavioral control
SARS: Severe Acute Respiratory Syndrome
SN: Subjective norm
TPB: Theory of planned behavior
